# The filling factor of the sEMG signal at low contraction forces in the quadriceps muscles is influenced by the thickness of the subcutaneous layer

**DOI:** 10.3389/fphys.2023.1298317

**Published:** 2024-01-05

**Authors:** Javier Rodriguez-Falces, Armando Malanda, Cristina Mariscal, Javier Navallas

**Affiliations:** ^1^ Department of Electrical and Electronical Engineering, Public University of Navarra, Pamplona, Spain; ^2^ Department of Clinical Neurophysiology, Hospital Complex of Navarra, Pamplona, Spain

**Keywords:** surface EMG, filling factor, form factor, interference pattern analysis, probability density function (PDF), sEMG RMS amplitude, subcutaneous layer

## Abstract

**Introduction:** It has been shown that, for male subjects, the sEMG activity at low contraction forces is normally “pulsatile”, i.e., formed by a few large-amplitude MUPs, coming from the most superficial motor units. The subcutaneous layer thickness, known to be greater in females than males, influences the electrode detection volume. Here, we investigated the influence of the subcutaneous layer thickness on the type of sEMG activity (pulsatile vs. continuous) at low contraction forces.

**Methods:** Voluntary surface EMG signals were recorded from the *quadriceps* muscles of healthy males and females as force was gradually increased from 0% to 40% MVC. The sEMG filling process was examined by measuring the EMG filling factor, computed from the non-central moments of the rectified sEMG signal.

**Results:** 1) The sEMG activity at low contraction forces was “continuous” in the VL, VM and RF of females, whereas this sEMG activity was “pulsatile” in the VL and VM of males. 2) The filling factor at low contraction forces was lower in males than females for the VL (*p* = 0.003) and VM (*p* = 0.002), but not for the RF (*p* = 0.54). 3) The subcutaneous layer was significantly thicker in females than males for the VL (*p* = 0.001), VM (*p* = 0.001), and RF (*p* = 0.003). 4) A significant correlation was found in the vastus muscles between the subcutaneous layer thickness and the filling factor (*p* < 0.05).

**Discussion:** The present results indicate that the sEMG activity at low contraction forces in the female quadriceps muscles is “continuous” due to the thick subcutaneous layer of these muscles, which impedes an accurate assessment of the sEMG filling process.

## 1 Introduction

The process by which the surface EMG signal is filled up with motor unit potentials (MUPs) as contraction force is gradually increased is a process governed by motor unit recruitment schemes and rate coding strategies (Navallas et al., 2023). Knowledge on the process of filling of the sEMG signal is valuable in several areas, such as detection of motor unit (MU) loss and reinnervation (Nandedkar et al., 2020), prosthesis control (Farina et al., 2010), fatigue monitoring (Holtermann et al., 2009), and also to separate motions (Nazarpour et al., 2007) and contraction levels (Hussain et al., 2009). However, the sEMG filling process has not yet been characterized in detail and many aspects of this process remain unresolved, as explained below. This is partly due to the fact that the sEMG characteristics during the filling process depend not only on neural factors, but also on the anatomical specificities of the muscle (such as thickness of the subcutaneous layers, arrangement of muscle fibers, etc.) and the spatial organization of motor units within the muscle (Rodriguez-Falces et al., 2023).

**FIGURE 1:**
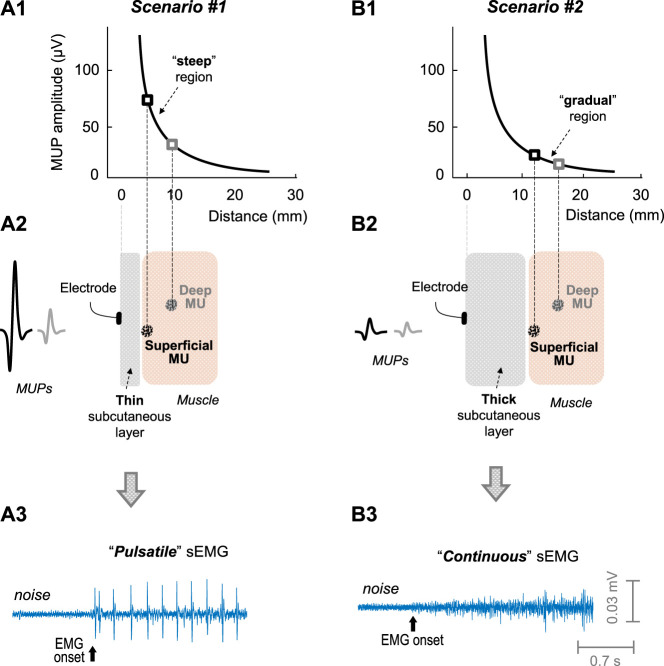
Impact of the subcutaneous layer thickness on the sEMG activity. Scenarios #1 (left) and #2 (right) represents muscles with a thin and thick subcutaneous layer, respectively. **(A1 and B1)**—Decline of the amplitude of a motor unit potential (MUP) with MU-to-electrode distance, according to the measurements of Roeleveld et al. (1997). In scenario #1 (plot **A2**, thin subcutaneous layer), the motor unit located superficially in the muscle would generate a MUP much larger than that located at deeper regions: this scenario would lead to a “pulsatile” sEMG activity (plot **A3**). In scenario #2 (plot **B2**, thick subcutaneous layer), there would be slight differences in MUP amplitude between the motor units located superficial and deep in the muscle (plot **B2**): this scenario would lead to a “continuous” sEMG activity (plot **B3**).

A condition necessary to study the filling process of the sEMG signal is that, at the beginning of this filling process, the sEMG activity must be “pulsatile” (see Figure 1A3), i.e., composed of a few large-amplitude MUPs, clearly standing out from background noise (Rodriguez-Falces et al., 2023). Only if the sEMG activity at low force levels is pulsatile, the successive activation of additional motor units with increasing contraction force will progressively fill up the baseline with MUPs, until this baseline is completely obscured, thus giving the opportunity to examine the filling process in a comprehensive manner. However, this initial “pulsatile condition” is not fulfilled in all muscles. In fact, in our previous study, we found that the initial sEMG activity was pulsatile for most of the *vastus lateralis* and *medialis* examined (75% and 87%, respectively), but not for the *rectus femoris* (3%) (Rodriguez-Falces et al., 2023). Interestingly, the *rectus femoris* sEMG signal contained many MUP discharges of small amplitude, which overlapped extensively, i.e., a signal which looks “continuous” (see Figure 1b3).

The striking differences in the initial sEMG activity between the *vastus* muscles and the *rectus femoris* could be related to the fact that the thickness of the subcutaneous layer is significantly lower in the *vastus* muscles (∼5 mm) compared to the *rectus femoris* (∼9 mm) (Caresio et al., 2015). In fact, the thickness of the subcutaneous layer determines the number of fibers significantly contributing to the sEMG signal (Farina and Rainoldi, 1999). The rationale for this is that the amplitude of a MUP decreases exponentially (and not linearly) as the distance from the motor unit to the recording electrode increases (Roeleveld et al., 1997), as shown in plots a1 and a2 of Figure 1. Due to this exponential decline, the curve of MUP amplitude with MU-to-electrode distance can be roughly divided into two separate regions: a “steep” region where MUP amplitude decreases rapidly with MU-to-electrode distance (plot a1), and a “gradual” region where the curve flattens (plot b1). Based on this, when the muscle subcutaneous layer is thin (plot a2 in Figure 1), those MUs located superficially in the muscle would fall in the “steep” region of the curve, and, therefore, would generate MUPs much larger in amplitude compared to MUs located deeper in the muscle, thus favouring the appearance of a “pulsatile” sEMG activity (plot a3). Conversely, when the muscle subcutaneous layer is thick (plot b2), all MUs would fall in the “gradual” region of the curve, and, therefore, superficial and deep MUs would generate MUPs with small differences in amplitude, thus favouring the appearance of a “continuous” sEMG activity (plot b3).

If the presence of pulsatile sEMG activity at low contraction forces is more easily found in muscles with thin subcutaneous layers, then one would expect to find a higher percentage of initially-pulsatile sEMG activity in males than in females, given the thicker subcutaneous layers in the latter (Caresio et al., 2015). In our previous study, the filling process of the sEMG signal was investigated in the quadriceps muscles only for healthy young males (Rodriguez-Falces et al., 2023). Therefore, it is yet to be determined how the filling process of the sEMG signal would be in these same muscles for healthy young females. Would females show initially-pulsatile sEMG activity in the *vastus lateralis* and *medialis* as males do?

The objectives of the present study were: (1) to examine the type of sEMG activity (pulsatile vs. continuous) of the quadriceps muscles of males and females at low contraction forces, and to relate this myoelectric activity with the subcutaneous layer thickness, and (2) to determine whether it is possible to characterize the sEMG filling process in the females’s *quadriceps* muscles as contraction force was gradually increased. Since the subcutaneous layer of the female quadriceps muscles is noticeably thicker compared to that of men (Caresio et al., 2015), it is hypothesized that the sEMG activity at low contraction intensities would be “continuous” in these female muscles, and thus it would not be possible to assess the sEMG filling process in such muscles.

The present objectives will be addressed using a new analysis tool called the filling factor, which is derived from the probability density function (PDF) of the sEMG amplitudes factor (Navallas et al., 2023). The filling factor allows to quantify the degree to which an EMG signal has been filled, and thus has the potential to characterize the EMG filling process, and also to differentiate between a “pulsatile” and a “continuous” sEMG activity (Rodriguez-Falces et al., 2023).

## 2 Material and methods

### 2.1 Participants

G-Power software was used to calculate sample size in accordance to Beck (2013). The software indicated that, for a statistical power (1-β) of 0.90, a sample size of 15 participants per group was needed. Thirty-five participants aged between 20 and 28 years, 18 males (mean age ±SD: 22 ± 3 years) and 16 females (21 ± 2 years), volunteered to participate in this study. The average height and body mass for males were 181 ± 5 cm and 73 ± 4 kg, respectively, whereas for females these measures were 170 ± 4 cm and 59 ± 4 kg, respectively. All participants provided written informed consent before the experimental session. None of the participants reported any neuromuscular or sensory impairments at least 6 months prior to the study. The experiments were approved by the Ethics Committee Board of the Public University of Navarra, Spain (PI-010/21), and conducted following the guidelines of the Declaration of Helsinki.

### 2.2 Experimental setup and force recording

All measurements were conducted on the quadriceps muscle while participants gradually increased the isometric knee extension force. To do so, participants were comfortably seated on a custom-built chair, with a trunk-thigh angle of 100°, and the knee joint angle at 90°. Potential upper body movements were limited by securing participants to the seat with two crossed shoulder harnesses and a belt on the lower abdomen. Quadriceps force was recorded during the gradually increasing isometric contractions using a strain gauge (STS, SWJ, China, sensitivity 2 mV/V and 0.0017 V/N, linear range: 0–2452 N) that was attached to the chair and securely strapped to the ankle with a custom made mould. The force signal (from the isometric knee extension) was digitized at 1,000 Hz using an analog/digital converter (MP150; BIOPAC, Goleta, CA, United States).

### 2.3 Localization of the innervation zone and the muscle fibers’ direction

The innervation zone location and the muscle fibers’ direction were identified in each muscle using a dry linear array of 16 electrodes (5 mm inter-electrode distance) during gentle isometric contractions. The linear array was connected to a multichannel amplifier (OT Bioelettronica, Torino; bandwidth 10–500 Hz). EMG signals were registered under single-differential (bipolar) mode. The location of the innervation zone was that corresponding to the channel of the array showing minimum amplitude or phase reversal (Rodriguez-Falces and Place, 2017). The direction of the muscle fibers was determined by choosing the orientation of the array that yielded the clearest propagation of action potentials between the innervation zone and tendon regions (Farina et al., 2002).

### 2.4 Electromyographic recordings

Electromyographic potentials were registered simultaneously from the *vastus lateralis* (VL), *vastus medialis* (VM), and *rectus femoris* (RF) muscles. EMG potentials were recorded using self-adhesive electrodes (Ag/AgCl, Kendall Meditrace 100), with a circular shape (recording diameter, 10 mm). In each muscle, a pair of electrodes were placed in bipolar configuration, with an inter-electrode distance of 20 mm. In the VL and VM, the bipolar electrodes were placed along a line parallel to the direction of the muscle fibers, with the proximal electrode of the pair located over the innervation zone, as performed previously (Rodriguez-Falces and Place, 2017). In the RF, the electrodes were positioned lengthwise over the muscle belly. The “ground” electrode was placed over the patellar tendon. Surface EMG signals were amplified (gain, 500 V/V), filtered with a bandpass filter (10–500 Hz), and digitized (sampling frequency of 5,000 Hz) using an analog/digital converter (MP150; BIOPAC, Goleta, CA). With respect to the maximum acceptable noise level, the criterion was that p2p noise should remain below 5–6 μV throughout the experimental session (Tankisi et al., 2020).

### 2.5 Experimental protocol

The participants were well familiarized with performing isometric knee extension contractions, since they all have been involved in previous experiments. The participants were instructed not to exercise with their legs or to engage in heavy physical work for a day beforehand. On the experiment day, participants were allowed to complete several warm-up contractions.

The experiment began with the participants performing 3 brief (4s) control maximum voluntary contractions (MVCs), with 3 min of rest in between. The peak forces from these 3 MVCs were averaged to determine the MVC force. This reference maximal force was used to determine the force level corresponding to 40% MVC.

After the MVC contractions, 5 minutes of rest were provided. Thereafter, to examine the sEMG filling process, an isometric knee extension “ramp” contraction was performed, in which force was increased linearly from 0 up to 40% of MVC force in 60s. The desired force trajectory was displayed on a computer screen in front of the participant. The rate of force increase during the ramp contraction was deliberately slow so that the process of EMG filling could be studied in detail, especially during the initial part of the contraction (15s to reach 10% MVC). The choice of the 40% MVC as the upper force limit was made for two reasons: 1) it ensured the sEMG interference pattern to be completely formed (Bril and Fuglsang-Frederiksen, 1984), and 2) the potential effect of fatigue is limited, especially during the first half of the contraction (Rodriguez-Falces and Place, 2021).

### 2.6 Measurement of subcutaneous layer thickness

All measurements were performed using an ultrasound device (SonoScape E2 Pro), which was equipped by a linear-array transducer (code L745) with variable frequency band (6.0–12.2 MHz). Gain was fixed at 50% of the range. The depth setting was set at 50 mm. Pictures were stored and transferred to a computer for processing. Thickness measures were performed using the image processing program ImageJ (National Institutes of Health, Bethesda, MD, United States). Specifically, the subcutaneous layer thickness was measured as the perpendicular distance (mm) between the bottom of the skin and the adipose tissue/muscle interface (Caresio et al., 2015). For each muscle, three images were acquired, and three measurements per image were made. Thus, a total of nine measurements per muscle were extracted and averaged to yield the subcutaneous layer thickness. The ultrasound transducer was placed at the same position where the electrodes were originally located (after these electrodes had been removed).

### 2.7 Definition of the EMG filling factor

The filling factor is an index of the degree to which an EMG signal has been filled, and thus serves to characterize the EMG filling process (Navallas et al., 2023). The filling factor is intrinsically related to the shape of the sEMG PDF distribution (Navallas et al., 2023). Specifically, this index is calculated from the first two non-central of the rectified sEMG signal, which are defined as follows:

$$m_{1}=\frac{1}{N}\sum_{n=0}^{N-1}\left|x\left[n\right]\right|$$

$$m_{2}=\frac{1}{N}\sum_{n=0}^{N-1}{\left|x\left[n\right]\right|}^{2}$$

where x[n] is the sampled sEMG signal and N is the number of samples in each segment of recording. Note that *m*_1_ represents the rectified sEMG mean, whereas *m*_2_ is the square of the sEMG root mean square (RMS). Then, the EMG filling factor (FF) is calculated as the ratio between *m*_1_^2^ and *m*_2_ as follows:

$$FF=\frac{{m_{1}}^{2}}{m_{2}}$$

### 2.8 Analysis of the sEMG filling process with the filling factor

To analyze the sEMG filling process, the first step was to calculate the filling factor over successive non-overlapping windows of the sEMG signal. Window duration was set at 0.7 s, similar to our previous report (Rodriguez-Falces et al., 2023). The second step was to plot the filling factor values as a function of the rectified sEMG mean (i.e., the above-defined m_1_), as done previously by other authors (Nandedkar et al., 2020; Navallas et al., 2023). The reasons why the filling factor was examined against sEMG mean, instead of against force, were as follows. First, force recording increases the time and complexity of the experimental set-up, a critical aspect in the clinical environment. Besides, changes in the rectified sEMG mean parallels those in force (Nandedkar et al., 2004). Nonetheless, in the present study force was also recorded to ensure the same force profile in all participants.

Another goal of the study was to determine how often the sEMG activity at low contraction forces was “pulsatile” and “continuous” in the male and female muscles. The filling factor threshold used to differentiate between “pulsatile” and “continuous” sEMG activity was 0.45, as previously established (Rodriguez-Falces et al., 2023): below this threshold, the sEMG was considered pulsatile. The filling factor “at low contraction forces” was calculated as the average of the filling factors from the first four 0.7-s epochs of the sEMG signal (∼ first 3s).

To evaluate the feasibility to analyze the sEMG filling process, we examined whether, as force was gradually increased, the successive incorporation of MUPs to the sEMG signal made the filling factor increase within a wide range. To do this, the total variation of the filling factor throughout the 40-s ramp contraction was assessed.

Two possible effects of the subcutaneous layer thickness were assessed. First, as theorized in the introduction, muscles with thinner subcutaneous layers would more likely present a pulsatile sEMG activity at low contraction forces, compared to muscles with thicker layers. In this line, the possible relation between the subcutaneous layer thickness and the filling factor value “at low contraction forces” was explored. In addition, the subcutaneous layer attenuates the sEMG signal (Ptaszkowski et al., 2019), and hence it would be expected that those individuals with thinner subcutaneous layers would present higher values of sEMG amplitude compared to individuals with thicker layers. In this line, the possible relation between the subcutaneous layer thickness and sEMG RMS amplitude at low contraction forces was examined.

### 2.9 Statistics

Kolmogorov-Smirnov tests confirmed that each variable analyzed in the current study was normally distributed. To examine differences in the filling factor values at low contraction forces between males and females, a two-way ANOVA (gender × muscle) was performed. Similarly, a two-way ANOVA was conducted to investigate possible differences in the sEMG RMS amplitude at low contraction forces between males and females (gender × muscle). When main effects or interactions were significant, Student-Newman-Keuls *post hoc* tests were conducted. Pearson’s correlation (R) was used to examine the relation between the subcutaneous layer thickness and the filling factor at low contraction forces, as well as the relation between the subcutaneous layer thickness and sEMG RMS amplitude. The interpretations of correlations were based on the criteria set by Cohen (1988), with a modified terminology, i.e., >0.5 as good, 0.3–0.5 as moderate, and <0.3 as poor. Statistical significance was set at *p* < 0.05. All the results were expressed as mean ± standard deviation (SD) unless differently stated.

## 3 Results

### 3.1 Representative examples of the sEMG signal at the beginning of the contraction in males and females

**FIGURE 2:**
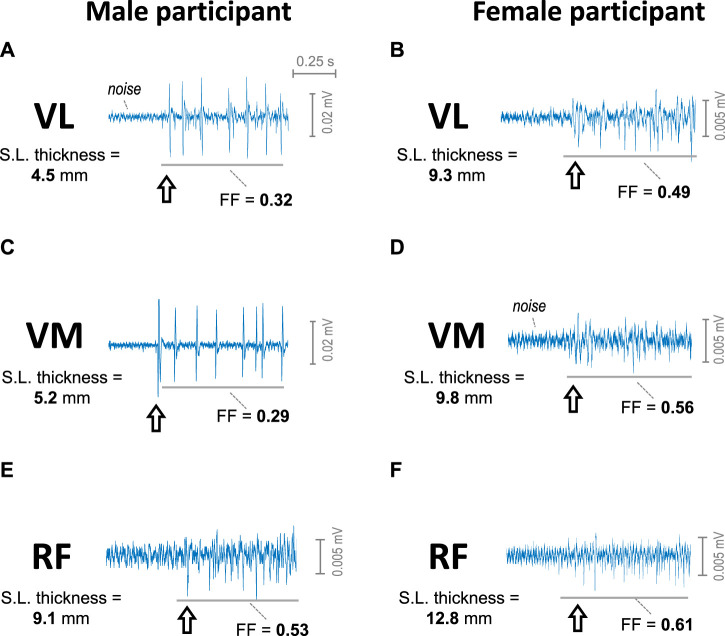
Representative examples of the sEMG signal at the beginning of the contraction as force was gradually increased from 0 in the vastus lateralis **(A, B)**, vastus medialis **(C, D)**, and rectus femoris **(E, F)** of a male participant (first column) and of a female participant (second column). The arrow indicates the onset of the sEMG activity. The subcutaneous layer thickness of each muscle is indicated at the left of each plot. The filling factor (FF) of a 0.7-s segment of the sEMG signal is indicated below each trace.

Figure 2 shows representative examples of a short (∼1 s) segment of the sEMG signal recorded at the beginning of the contraction from VL, VM, and RF muscles of a male participant and a female participant as contraction force was gradually increased from zero force. It can be seen that, in the VL and VM of the male participant (plots a and c), the initial sEMG activity contained a few large-amplitude MUPs, clearly standing out from noise (i.e., pulsatile activity). In contrast, in the VL, VM and RF of the female participant (plots b, d, and f) and the RF of the male subject (plot e), the initial sEMG activity was formed by many small-amplitude MUPs, which hardly stood out from noise (i.e., continuous activity). Noteworthy, the subcutaneous layer thickness of the VL and VM of the male participant (about 5 mm) had much lower values compared to the other muscles (between 9 and 13 mm). Note also that the filling factor (FF) values of the VL and VM of the male participant were below 0.45, whereas in the other muscles, the filling factor values exceeded the threshold of 0.45.

### 3.2 Representative examples of the sEMG filling process in males and females

**FIGURE 3:**
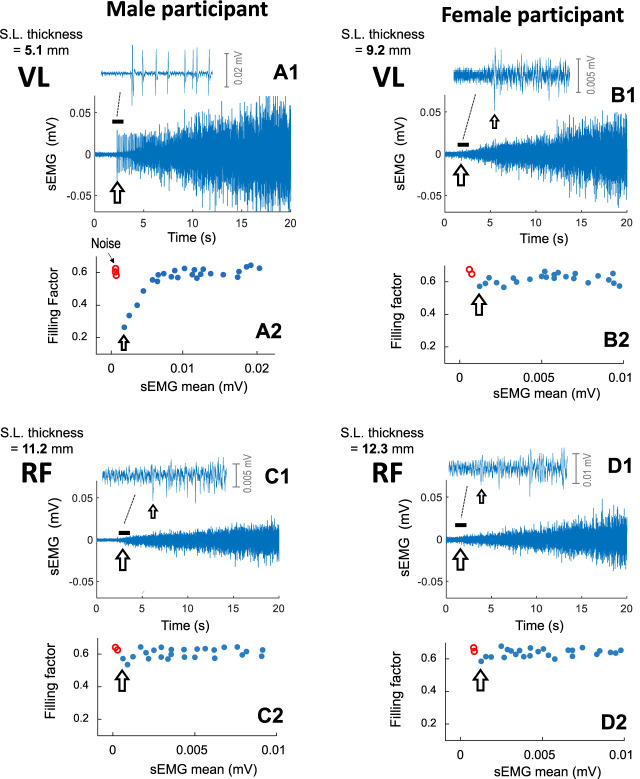
Representative examples of the sEMG filling process in the vastus lateralis **(A1, B1)** and rectus femoris **(C1, D1)** of a male participant (first column) and of a female participant (second column), as force was gradually increased from 0% to 40%. For each muscle, a short (∼1 s) segment of the sEMG signal at the onset of the contraction is depicted at the top, whereas the filling factor values are shown at the bottom **(A2, B2, C2, and D2)**. The subcutaneous layer thickness of each muscle is indicated at the top left of each plot. For visualization purposes, only the first 20 s of the contraction was shown. The arrow indicates the onset of the sEMG activity.

Figure 3 shows typical examples of sEMG signals recorded from the VL and RF of a male participant (first column) and of a female participant (second column), as force was gradually increased from 0% to 40%. For each participant, a detail of the initial sEMG activity is depicted at the top (first row), and the filling factor values extracted from the sEMG signal are shown at the bottom (third row).

In the VL of the male participant (plot a1), the initial sEMG activity contained a few large-amplitude MUP spikes. The abrupt onset of these large-amplitude MUPs provoked a prominent abrupt fall in the filling factor to ∼0.39 (see the grey arrow in plot a2). After this “abrupt onset”, as contraction force was increased, the successively recruited MUPs gradually filled the sEMG signal, and the filling factor increased within a wide range, from 0.39 to ∼0.63 (plot c.3).

In contrast, in the RF of the male participant (plot c1) and also in the VL and RF of the female participant (plots b1 and d1), the initial sEMG activity was “continuous”, consisting of many small-amplitude MUPs with great overlap between them (see the insets in these plots). After this “continuous onset”, as force was increased, the filling factor did not increase, but rather fluctuated around 0.63 (see plots c2, b2, and d2).

To better appreciate the differences in sEMG amplitude, the same voltage scale in the (sEMG axis) was deliberately utilized in all cases. Note that the, in the VL of the male subject (plot a1), the sEMG amplitude was markedly higher than in the other muscles shown in Figure 3 (plots c1, b1, and d1).

### 3.3 Group analysis of the sEMG filling factor, RMS amplitude and subcutaneous layer thickness

In the VL and VM of males, the initial sEMG activity was “pulsatile” for the majority of subjects (76% and 88%, respectively, Table 1), whereas, in females, only a few cases of initially-pulsatile sEMG activity was found in these muscles (19% and 12%, respectively). However, in the RF of both males and females, the initial sEMG activity was “continuous” for most subjects (Table 1).

**TABLE 1:** Top–Percentage of subjects in which the initial activity was pulsatile vs. continuous in the vastus lateralis (VL), vastus medialis (VM), and rectus femoris (RF) in male and female participants.

	*VL*		*VM*		*RF*	
	*Males*	*Females*	*Males*	*Females*	*Males*	*Females*
% of subjects with an initial “pulsatil” sEMG activity	78% (14 of 18)	19% (3 of 16)	89% (16 of 18)	12% (2 of 16)	6% (1 of 18)	0%
% of subjects with an initial “continuous” sEMG activity	22% (4 of 18)	81% (13 of 16)	11% (2 of 18)	81% (13 of 16)	94% (17 of 18)	100%
Filling factor during the first 3s of the contraction	0.40 ± 0.08	0.54 ± 0.05 *	0.39 ± 0.08	0.53 ± 0.06 *	0.53 ± 0.07	0.56 ± 0.05
Total variation of the filling factor	0.21 ± 0.05	0.08 ± 0.02 *	0.22 ± 0.05	0.07 ± 0.02 *	0.04 ± 0.01	0.03 ± 0.01
sEMG RMS during the first 3s of the contraction (μV)	4.9 ± 1.7	3.6 ± 1.1 *	4.6 ± 1.4	3.5 ± 1.0 *	3.6 ± 0.9	3.4 ± 0.9
Subcutaneous layer thickness (mm)	5.0 ± 1.3	10.3 ± 2.3 *	5.7 ± 1.4	11.3 ± 2.3 *	8.7 ± 2.3	12.3 ± 2.2 *

Bottom–Mean ± SD values of the filling factor and sEMG RMS amplitude during the first 3s of the contraction for the VL, VM, and RF in male and female participants. Mean ± SD values of the subcutaneous layer thickness are also shown. * indicates significant difference between males and females.

**FIGURE 4:**
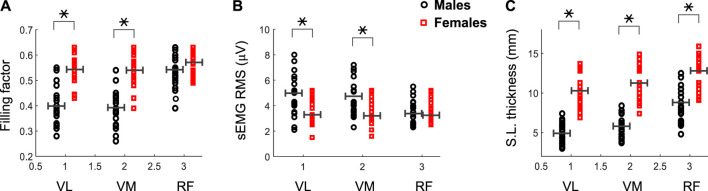
Mean and individual values of the filling factor **(A)** and sEMG RMS amplitude **(B)** during the first 3 s of the contraction for the vastus lateralis (VL), vastus medialis (VM) and rectus femoris (RF) of male and female participants. Data of the subcutaneous layer thickness are also shown **(C)**. Means are shown as horizontal lines. * indicates significant difference between males and females.

The filling factor during the first 3s of the contraction was significantly lower in males than females for the VL (0.40 vs. 0.54, *p* = 0.003) and VM (0.39 vs. 0.53, *p* = 0.002) muscles (see Figure 4A and Table 1), whereas this index had similar values for the two genders in the RF muscle (0.53 vs. 0.56, *p* = 0.54). A significant gender × muscle interaction was found for the filling factor (*p* = 0.03). The sEMG RMS amplitude at low contraction forces was significantly greater in males than females for the VL (4.9 vs. 3.6 μV, *p* = 0.02) and VM (4.6 vs. 3.5 μV, *p* = 0.01) muscles (Figure 4B; Table 1), but this amplitude was comparable for the two genders in the RF muscle (3.6 vs. 3.4 μV, *p* = 0.47). There was a significant gender × muscle interaction for the sEMG RMS (*p* = 0.01). The subcutaneous layer was significantly thicker for females compared to males in the VL (5.0 vs. 10.3 mm, *p* = 0.001) and VM (5.7 vs. 11.3 mm, *p* = 0.001), and also in the RF (8.7 vs. 12.3 mm, *p* = 0.003), as shown in Figure 4C and Table 1.

### 3.4 Correlation analysis

**FIGURE 5:**
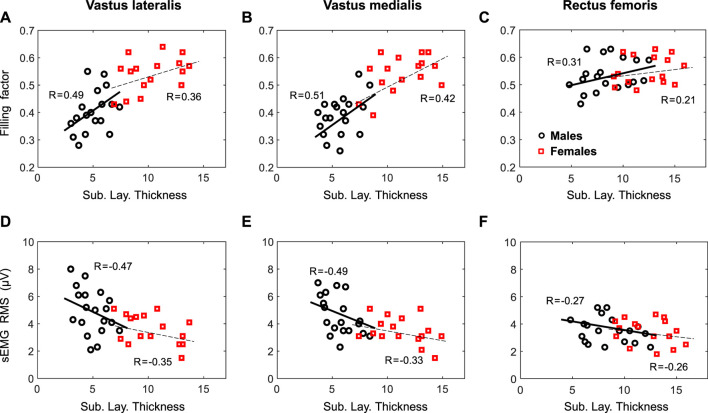
First row—Relationship by gender between the filling factor measured at low contraction forces and the subcutaneous layer thickness for the vastus lateralis **(A, D)**, vastus medialis **(B, E)**, and rectus femoris **(C, F)**. Second row—Relationship by gender between the sEMG RMS and the subcutaneous layer thickness for the same muscles. Regression coefficients, R, are included for each plot.

At group level, the correlation between the filling factor and the subcutaneous layer thickness was strong (*p* <0.001) for the VL (r = 0.69) and VM (r = 0.71), but weak for the RF (r = 0.30, *p* = 0.06), as shown in Figures 5A–C. At gender level, a moderate correlation was found for the VL of males (r = 0.49, *p* = 0.01), but a weak correlation for females (r = 0.36, *p* = 0.03). For the VM, moderate correlations were found for both males (r = 0.51, *p* = 0.01) and females (r = 0.42, *p* = 0.02). In the case of the RF, there were weak correlations for males (r = 0.31, *p* = 0.06), and females (r = 0.21, *p* = 0.13).

At group level, the regression analysis showed a strong correlation (*p* <0.001) between sEMG RMS amplitude and the subcutaneous layer thickness for the VL (r = −0.61), VM (r = −0.59), but a weak correlation for the RF (r = −0.25, *p* = 0.08), as depicted in Figures 5D–F. When separated by gender, a moderate correlation was found for the VL of both males (r = −0.47, *p* = 0.02), and females (r = −0.35, *p* = 0.04). Similarly, a moderate correlation (*p* <0.05) were found for the VL of both males (r = −0.49, *p* = 0.01), and females (r = −0.33, *p* = 0.04). In the case of the RF, there were weak correlations for males (r = −0.27, *p* = 0.07) and females (r = −0.26, *p* = 0.12).

## 4 Discussion

The main findings of the present study were the following:

(1) As quadriceps force was gradually increased, the sEMG activity at the beginning of the contraction was “continuous” in the VL and VM of females, but “pulsatile” in the VL and VM of males. In the RF, the initial sEMG activity was “continuous” for both males and females.
(2) The filling factor at low contraction forces was lower in males than females for the VL and VM, but not for the RF.
(3) The sEMG RMS amplitude was higher in males than females for the VL and VM, but not for the RF.
(4) The thickness of the subcutaneous layer was significantly greater in females than males for the VL, VM and RF muscles.
(5) Significant correlations were found in the vastus muscles between the filling factor and the subcutaneous layer thickness, and between the sEMG RMS and the subcutaneous layer thickness.

### 4.1 Different type of sEMG activity at low contraction forces in males and females

We found that, in the *vastus lateralis* and *medialis* of females, the sEMG activity at the beginning of the contraction generally contained many MUP discharges of small amplitude, which overlapped extensively, so that the signal looked “continuous”. In contrast, we observed that, in the *vastus* muscles of males, the initial sEMG activity was mainly “pulsatile”, that is, formed by a few large-amplitude spikes, clearly standing out from noise. This striking difference in the type of sEMG activity at low contraction forces between males and females (pulsatile vs. continuous) is thought to be due to the differences in the subcutaneous layer thickness between the two genders. Indeed, we found that, in the *vastus* muscles, the subcutaneous layer was remarkably thinner in males (5.0, 5.7 mm) than in females (10.3, 11.3 mm), in agreement with Caresio et al. (2015) and Paris et al. (2022). We propose that the thickness of the subcutaneous layer is a major determinant for the type of sEMG activity at low contraction forces. The rationale is based on the exponential decline of MUP amplitude with MU-to-electrode distance, as depicted in Figure 1 (Roeleveld et al., 1997). Accordingly, in those muscles with a “thin” subcutaneous layer, such as the *vastus* muscles of males, (scenario #1 in Figure 1), superficial motor units would generate MUPs with much greater amplitude than deep motor units, thus favoring the appearance of a “pulsatile” sEMG activity. In contrast, when the subcutaneous layer is “thick” (scenario #2 in Figure 1), there would be only moderate differences in MUP amplitude between superficial and deep motor units: this scenario promotes the generation of a continuous sEMG activity. This reasoning and the present findings are in line with the studies of Farina and others, who found that an increase in subcutaneous layer thickness produces an increase in the detection volume area (Farina and Rainoldi, 1999), which allows that a higher number of electrical generators (muscle fibers) contribute to the recording electrodes (Farina et al., 2002), thus leading to a sEMG signal with a high overlapping between discharges, i.e., a continuous sEMG signal.

Contrary to the results in the *vastus* muscles, the initial sEMG activity in the *rectus femoris* was essentially “continuous” for both males and females. The similar type of sEMG activity in males and females may appear surprising in view of the fact that the subcutaneous layer thickness of the *rectus femoris* was lower in males (8.7 mm) than females (12.3 mm). Moreover, we found that in the case of males, “modest” skinfold values (between 5 and 8 mm) exhibits a wide variety of filling factor values, ranging from 0.45 to 0.63, which essentially correspond to a continuous sEMG activity. These high values of the filling factor may be due to the complex anatomic features of the *rectus femoris*, which is known to have a muscle-within-a-muscle configuration in which an outer unipennate muscle surrounds an inner bipennate muscle (Kassarjian et al., 2014). With so complex fiber arrangement, including various layers of fibers, it can occur that for some individuals the sEMG activity at low contraction forces shows many MUPs with a great overlap between them (i.e., a continuous pattern), even with modest skinfold values. Even so, we found that for the male *rectus femoris*, a moderate level of association exists between the filling factor and skinfold thickness (r = 0.31, *p* = 0.06). For the *rectus femoris* of females the average skinfold thickness was very high (∼12.3 mm), and thus one would expect to find a clear “continuous” sEMG activity in most subjects, with filling factor values above 0.5, as indeed was the case. Consistent with this, we found no correlation between the filling factor and skinfold thickness for the female *rectus femoris* (r = 0.21, *p* = 0.13). The explanation for this lack of correlation could be due to the fact that, once the skinfold thickness exceeds a certain limit, say 10 mm, there would be little difference in “how continuous” the sEMG signal is, and all filling factor values would be above 0.5.

### 4.2 The filling factor as a tool to differentiate between continuous and pulsatile sEMG activity

Classification of the sEMG activity as “pulsatile” or “continuous” has been normally made by visual inspection of the signal, and thus it is subjected to the subjectivity of individual interpretation (Nandedkar et al., 2020). Here, we proposed that such classification can be made on the basis of the filling factor index, and more specifically, choosing a filling factor threshold of 0.45 to make such differentiation. The appropriateness of the 0.45 threshold was corroborated by visual inspection: in those muscles where the sEMG activity contained a few large-amplitude MUP spikes, clearly standing out from background activity, the filling factor was always below 0.45 (this occur in >78% of the cases in the VL and VM of males).

### 4.3 The filling process of the sEMG signal cannot be assessed in the female quadriceps muscles

Information about the sEMG filling process can only be obtained in scenarios where the successive incorporation of MUPs to the sEMG signal makes the filling factor increase within a wide range (Rodriguez-Falces et al., 2023). Because the highest limit of the filling factor is 0.63 (corresponding to a completely filled sEMG signal), the only way to achieve a wide range of variation for the filling factor is to ensure that the sEMG activity at the beginning of the contraction has a low filling factor value, say 0.3–0.4 (as in Figures 2C, 3A). In other words, the sEMG activity at low contraction forces must be pulsatile. However, we found that this condition was not satisfied in the female quadriceps muscles. Indeed, in these muscles, the initial sEMG activity was essentially continuous, with initial filling factor values generally mainly within the range 0.50–0.63, which hampered the possibility to study the sEMG filling process in detail. Clearly, with the highest filling factor being 0.63, if the initial filling factor values fall within the range 0.50–0.63, then as activation level was gradually increased, the filling factor either remains practically stable or increase within a narrow range. These conditions do not give the opportunity to accurately assess how the sEMG signal was progressively filled up with MUPs.

### 4.4 The thickness of the subcutaneous layer not only affect the amplitude of the sEMG signal, but also the type of sEMG activity (pulsatile vs. continuous)

It is known that the sEMG amplitude decreases as the distance between the recording electrode and the electrical generator (muscle fiber) increases (Ptaszkowski et al., 2019; Rodriguez-Falces et al., 2013). This is because the “average” tissue impedance increases with fiber-to-electrode distance (Petroksky, 2008). Moreover, the adipose tissue attenuates the EMG signal more markedly than muscle tissue and body fluid (Nordander et al., 2003). Therefore, the thicker the adipose layer, the greater the attenuation of the sEMG signal (Hemingway et al., 1995). Consistent with this, we found that sEMG RMS amplitudes were lower in the *vastus* muscles of females compared to those of males, due to the thicker subcutaneous layer of the former individuals. This adipose layer effect is further supported by the significative (negative) correlations found between the sEMG RMS amplitudes and the subcutaneous layer thickness.

One key finding of the present study is that the thickness of the subcutaneous tissue does not only work as a scale (attenuation) factor for the sEMG amplitude, but it also influences the type (composition) of the sEMG signal, at least for low contraction levels. Indeed, our thesis that the thickness of the subcutaneous tissue is a major determinant for the type of sEMG activity was substantiated by the good correlations found between the filling factor and the subcutaneous layer thickness. Indeed, we found that the initially-pulsatile sEMG activity (filling factor values below 0.45) was predominantly found in those quadriceps’ muscles with a thin adipose layer, i.e., the *vastus lateralis* and *vastus medialis*. On the contrary, initially-continuous sEMG activity (filling factor values above 0.45) was primarily found in the *rectus femoris*, known to have a thick subcutaneous layer. These results open a new perspective in the analysis of the surface EMG signal, as they underlie the idea that not only the amplitude of the surface EMG is important, but also the composition (type) of this signal is also informative: for example, whether the sEMG signal is pulsatile or continuous may provide information on the thickness of the adipose tissue.

### 4.5 Implications of the study

The present results have implications at several levels. The first issue refers to the assumption that, in healthy individuals, the sEMG activity is generally “continuous” at low contraction levels, as stated by Nandedkar et al. (2020). However, the current study indicates that the type of sEMG activity is essentially determined by the subcutaneous layer thickness. This means that factors affecting the adipose tissue, such as gender or muscle, could influence the type of myoelectric activity, and thus should be considered.

The present findings also have implications on the recent controversy on the accuracy of the Motor Unit Number Index (MUNIX) method (Bostock et al., 2019). Specifically, it has been shown that the MUNIX technique has low sensitivity to detect changes in motor unit size and MUP amplitude (Li et al., 2012). The reason is that, when MUPs overlap extensively at low contraction forces (i.e., continuous sEMG activity), information about motor unit size and number is lost, and MUNIX would depend only on CMAP parameters (Bostock et al., 2019). However, here we have shown that not all muscles generate continuous myoelectric activity at low forces: indeed, in the case of the *vastus* muscles of males, the initial sEMG activity is predominately pulsatile. Therefore, the above limitation of the MUNIX method would only apply on those muscles that generate continuous myoelectric activity at low contraction forces.

The present results would also be relevant for prosthesis control, and more specifically for the accurate determination of the onset of myoelectric activity (Bonato et al., 1998). Indeed, any method aimed at discriminating the sEMG signal from background noise would have better results in muscles with a pulsatile myoelectrical activity, due to the high signal-to-noise (SNR) ratio of this signal (Vannozzi et al., 2010). Thus, attempts must be directed to identify those muscles in the forearm and legs with a higher predisposition to generate pulsatile sEMG activity. This initial “pulsatile condition” would be particularly important in those cases where muscle activation increases slowly, i.e., when SNR is low at the beginning of the contraction (Merlo et al., 2003).

### 4.6 Limitations in the analysis of the sEMG activity with low signal-to-noise ratio of the sEMG signal

It must be stressed that the SNR must be sufficiently high to be able to identify the type of sEMG activity, especially at the beginning of the contraction when the size of MUPs is generally small. Indeed, a low SNR makes it hard to discern the truth sEMG activity. In the present study, the lowest baseline noise levels measured were 2–3 uV (peak-to-peak), this value being similar for males and females. Considering a similar baseline noise level for males and females, the male subjects would generally present higher SNR values than females at low contraction levels, as the size of the male MUPs is normally greater than female MUPs due to the thicker subcutaneous layer of the latter. In other words, female subjects present lower SNR values (compared to males) due to the greater attenuation of MUPs caused by their thick subcutaneous layer.

In the present study we have observed that, at low force levels, the MUPs of female muscles were comparable in magnitude to baseline noise amplitude (Figure 2). Therefore, the truth sEMG pattern of female muscles at low contraction forces is hard to be known, as this sEMG pattern is largely obscured/contaminated by the baseline noise. As a result, the continuous sEMG pattern observed in females is more due to the noise contamination than to the actual sEMG activity.

## 5 Conclusion

It has been demonstrated that, as quadriceps force was gradually increased, the sEMG activity at the beginning of the contraction was “continuous” in the female vastus muscles, whereas the initial sEMG activity was “pulsatile” in the corresponding male muscles. This difference in the type of sEMG activity at low contraction forces between males and females is most likely due to the differences in the subcutaneous layer thickness between the two genders (thicker in females than in males). Indeed, the initially-pulsatile sEMG activity was predominantly found in those quadriceps’ muscles with a thin adipose layer, whereas the initially-continuous sEMG activity was primarily found in muscles with a thick subcutaneous layer. The fact that the initial sEMG activity in the female quadriceps muscles was “continuous” impeded an accurate assessment of the sEMG filling process in this group.

The study also demonstrates that the filling factor is a useful method, not only to analyse the EMG filling process, but also to differentiate between a “continuous” and “pulsatile” sEMG activity.

The study opens a new perspective in the analysis of the surface EMG signal, as it underlies the idea that not only the amplitude of the sEMG is important, but also the type (composition) of signal (pulsatile vs. continuous) is informative, as it may provide valuable anatomical information of the muscle, such as the subcutaneous tissue thickness.

## Data Availability

The original contributions presented in the study are included in the article/supplementary material, further inquiries can be directed to the corresponding author.

## References

[B1] Beck T. W. (2013). The importance of *a priori* sample size estimation in strength and conditioning research. J. Strength Cond. Res. 27, 2323–2337. 10.1519/JSC.0b013e318278eea0 23880657

[B2] Bonato B. D'Alessio T. Knaflitz M. (1998). A statistical method for the measurement of muscle activation intervals from surface myoelectric signal during gait. IEEE Trans. Biomed. Eng. 45, 287–299. 10.1109/10.661154 9509745

[B3] Bostock H. Jacobsen A. B. Tankisi H. (2019). Motor unit number index and compound muscle action potential amplitude. Clin. Neurophysiol. 130 (9), 1734–1740. 10.1016/j.clinph.2019.05.031 31288985

[B4] Bril V. Fuglsang-Frederiksen A. (1984). Number of potential reversals (turns) and amplitude of the pattern of electrical activity of the abductor pollicis brevis muscle in patients with neurogenic diseases. Acta Neurol. Scand. 70, 169–175. 10.1111/j.1600-0404.1984.tb00816.x 6507030

[B5] Caresio C. Molinari F. Emanuel G. Minetto M. A. (2015). Muscle echo intensity: reliability and conditioning factors. Clin. Physiol. Funct. Imaging. 35 (5), 393–403. 10.1111/cpf.12175 24902991

[B6] Cohen J. (1988). Statistical power analysis for the behavioral sciences. Hillsdale: Laurence Erlbaum Associates, Inc.

[B7] Farina D. Cescon C. Merletti R. (2002). Influence of anatomical, physical, and detection-system parameters on surface EMG. Biol. Cybern. 86 (6), 445–456. 10.1007/s00422-002-0309-2 12111273

[B8] Farina D. Holobar A. Merletti R. Enoka R. M. (2010). Decoding the neural drive to muscles from the surface electromyogram. Clin. Neurophysiol. 121 (10), 1616–1623. 10.1016/j.clinph.2009.10.040 20444646

[B9] Farina D. Rainoldi A. (1999). Compensation of the effect of sub-cutaneous tissue layers on surface EMG: a simulation study. Med. Eng. Phys. 21 (6-7), 487–497. 10.1016/s1350-4533(99)00075-2 10624744

[B10] Hemingway M. A. Biedermann H. J. Inglis J. (1995). Electromyographic recordings of paraspinal muscles: variations related to subcutaneous tissue thickness. Biofeedback Self Regul. 20 (1), 39–49. 10.1007/BF01712765 7786926

[B11] Holtermann A. Grolund C. Karlsson J. S. Roeleveld K. (2009). Motor unit synchronization during fatigue: described with a novel SEMG method based on large motor unit samples. J. Electromyogr. Kinesiol. 19, 232–241. 10.1016/j.jelekin.2007.08.008 18207421

[B12] Hussain M. S. Reaz M. B. I. Mohd Yasin F. Ibrahimy M. I. (2009). Electromyography signal analysis using wavelet transform and higher order statistics to determine muscle contraction. Expert. Syst. 26 (1), 35–48. 10.1111/j.1468-0394.2008.00483.x

[B13] Kassarjian A. Rodrigo R. M. Santisteban J. M. (2014). Intramuscular degloving injuries to the rectus femoris: findings at MRI. AJR Am. J. Roentgenol. 202 (5), W475–W480. 10.2214/AJR.13.10931 24450607

[B14] Li X. Rymer W. Z. Zhou P. (2012). A simulation-based analysis of motor unit number index (MUNIX) technique using motoneuron pool and surface electromyogram models. IEEE Trans. Neural. Syst. Rehabil. Eng. 20 (3), 297–304. 10.1109/TNSRE.2012.2194311 22514208 PMC3556460

[B15] Merlo A. Farina D. Merletti R. (2003). A fast and reliable technique for muscle activity detection from surface EMG signals. IEEE Trans. Biomed. Eng. 50 (3), 316–323. 10.1109/TBME.2003.808829 12669988

[B16] Nandedkar S. D. Barkhaus P. E. Stålberg E. V. (2020). Form factor analysis of the surface electromyographic interference pattern. Muscle Nerve 62 (2), 233–238. 10.1002/mus.26922 32415859

[B17] Nandedkar S. D. Nandedkar D. S. Barkhaus P. E. Stalberg E. V. (2004). Motor unit number index (MUNIX). IEEE Trans. Biomed. Eng. 51, 2209–2211. 10.1109/TBME.2004.834281 15605872

[B18] Navallas J. Eciolaza A. Mariscal C. Malanda A. Rodriguez-Falces J. (2023). EMG probability density function: a new way to look at EMG signal filling from single motor unit potential to full interference pattern. IEEE Trans. Neur. Sys. Rehab. Eng. 31, 1188–1198. 10.1109/TNSRE.2023.3241354 37022369

[B19] Nazarpour K. Sharafat A. Firoozabadi S. (2007). Application of higher order statistics to surface electromyogram signal classification. IEEE Trans. Biomed. Eng. 54, 1762–1769. 10.1109/TBME.2007.894829 17926674

[B20] Nordander C. Willner J. Hansson G.-A. Larsson B. Unge J. Granquist L. (2003). Influence of the subcutaneous fat layer, as measured by ultrasound, skinfold calipers and BMI, on the EMG amplitude. Eur. J. Appl. Physiol. 89 (6), 514–519. 10.1007/s00421-003-0819-1 12712347

[B21] Paris M. T. Bell K. E. Avrutin E. Rosati K. Mourtzakis M. (2022). Influence of subcutaneous adipose tissue and skeletal muscle thickness on rectus femoris echo intensity in younger and older males and females. J. Ultrasound Med. 41 (9), 2355–2364. 10.1002/jum.15922 34921442

[B22] Petrofsky J. (2008). The effect of the subcutaneous fat on the transfer of current through skin and into muscle. Med. Eng. Phys. 30 (9), 1168–1176. 10.1016/j.medengphy.2008.02.009 18400550

[B23] Ptaszkowski K. Wlodarczyk P. Paprocka-Borowicz M. (2019). The relationship between the electromyographic activity of rectus and oblique abdominal muscles and bioimpedance body composition analysis - a pilot observational study. Diabetes Metab. Syndr. Obes. 12, 2033–2040. 10.2147/DMSO.S215982 31632113 PMC6789964

[B24] Rodriguez-Falces J. Malanda A. Mariscal C. Niazi I. K. Navallas J. (2023). Validation of the filling factor index to study the filling process of the sEMG signal in the quadriceps. J. Electromyogr. Kinesiol. 72, 102811. 10.1016/j.jelekin.2023.102811 37603990

[B30] Rodriguez-Falces J. Negro F. Gonzalez-Izal M. Farina D. (2013). Spatial distribution of surface action potentials generated by individual motor units in the human biceps brachii muscle. J. Electromyogr. Kinesiol. 23 (4), 766–777. 10.1016/j.jelekin.2013.03.011 23619102

[B25] Rodriguez-Falces J. (2017). A new method for the localization of the innervation zone based on monopolar surface-detected potentials. J. Electromyogr. Kinesiol. 35, 47–69. 10.1016/j.jelekin.2017.05.004 28595163

[B26] Rodriguez-Falces J. Place N. (2021). Sarcolemmal excitability, M-wave changes, and conduction velocity during a sustained low-force contraction. Front. Physiol. 12, 732624. 10.3389/fphys.2021.732624 34721063 PMC8554155

[B27] Roeleveld K. Stegeman D. F. Vingerhoets H. M. Van Oosterom A. (1997). Motor unit potential contribution to surface electromyography. Acta. Physiol. Scand. 160 (2), 175–183. 10.1046/j.1365-201X.1997.00152.x 9208044

[B28] Tankisi H. Burke D. Cui L. de Carvalho M. Kuwabara S. Nandedkar S. D. (2020). Standards of instrumentation of EMG. Clin. Neurophysiol. 131 (1), 243–258. 10.1016/j.clinph.2019.07.025 31761717

[B29] Vannozzi G. Conforto S. D'Alessio D. (2010). Automatic detection of surface EMG activation timing using a wavelet transform based method. J. Electromyogr. Kinesiol. 20, 767–772. 10.1016/j.jelekin.2010.02.007 20303286

